# First Reported Coexistence of Plasma Cell Gingivitis and Multiple Sclerosis: A Case Report

**DOI:** 10.1155/crid/2629260

**Published:** 2026-05-22

**Authors:** Hamid Tahernia, Bahareh Bagheri Todeshki, Mohammad Shahdadian, Amir Mansour Shirani

**Affiliations:** ^1^ Department of Oral Medicine, Isf.C, Islamic Azad University, Isfahan, Iran, khuisf.ac.ir; ^2^ Student Research Committee, Isfahan University of Medical Sciences, Isfahan, Iran, mui.ac.ir

**Keywords:** gingivitis, multiple sclerosis, plasma cell

## Abstract

**Background:**

Plasma cell gingivitis (PCG) is a rare, benign inflammatory condition of the oral mucosa characterized histologically by a dense plasmacytic infiltrate. Its etiology remains uncertain, with proposed triggers including allergens, infectious agents, and idiopathic immune reactions. Multiple sclerosis (MS), on the other hand, is a chronic autoimmune demyelinating disorder of the central nervous system that may indirectly influence oral health due to neurological impairment, altered immunity, or long‐term medication use. However, the coexistence of PCG and MS is extremely rare, and to the best of our knowledge, no such association has been previously documented in the literature. This report describes a unique case of PCG occurring in a patient with long‐standing, well‐controlled MS.

**Case Presentation:**

A 26‐year‐old woman with a 9‐year history of clinically stable and well‐controlled MS presented with complaints of gingival pain and spontaneous bleeding. Clinical examination revealed diffuse erythematous and edematous gingiva involving both anterior and posterior segments. Routine hematologic and biochemical investigations were unremarkable. An incisional biopsy of the affected gingiva was performed, and histopathological analysis demonstrated a dense, polyclonal plasma cell infiltrate consistent with PCG. Based on these findings, a diagnosis of PCG was established. The patient was initiated on a corticosteroid mouthwash regimen, resulting in symptomatic improvement. No systemic manifestations or MS exacerbations were noted during the course of dental evaluation and treatment. The patient was followed for 6mounths after initiating corticosteroid mouthwash. During this period, she demonstrated significant improvement of gingival erythema, with evidence of relapse. Her MS also remained clinically stable throughout the follow‐up.

**Conclusion:**

This case highlights the unusual coexistence of PCG and MS—an association that, to the best of our knowledge, has not previously been reported. While the relationship between the two conditions remains unclear, this observation raises the possibility of shared immunologic pathways or coincidental occurrence. Further studies are needed to explore potential connections and to guide clinicians in managing similar presentations.

## 1. Introduction

Plasma cell gingivitis (PCG) is an uncommon inflammatory disorder characterized by dense polyclonal plasma cell infiltration of the gingival connective tissue. Its etiology is most commonly linked to hypersensitivity reactions, and distinguishing PCG from conditions such as leukemia, mucous membrane pemphigoid, and lichen planus is essential [1]. This lesion is significant because it can mimic more serious disorders and result in severe gingival inflammation, pain, and bleeding. Prophylactic therapy has little effect on lesions. Since PCG shares clinically comparable pathologic features with leukemia, HIV infection, discoid lupus erythematosus, atrophic lichen planus, desquamative gingivitis, or cicatricial pemphigoid, which need to be distinguished by hematologic and serologic tests, early diagnosis is crucial [1, 2].

Multiple sclerosis (MS) is a chronic immune‐mediated demyelinating disease of the central nervous system. Immune dysregulation involving B cells, T cells, and macrophages plays a central role in its pathogenesis [3].

Although the exact pathogenesis of MS is not fully understood, several contributing factors have been identified. Genetic predisposition—particularly certain HLA alleles such as HLA‐DR2 and HLA‐DW2—along with environmental triggers, including viral infections, are believed to play significant roles in disease development [4, 5]. MS can cause optic neuritis, weakness and paresis, diplopia, ataxia, sphincter dysfunctions, constipation, depression, and many other signs and symptoms [6]. MS has four main progression patterns: relapsing‐remitting, secondary progressive, primary progressive, and progressive relapsing [7]. The main treatment options for acute attacks are corticosteroids; however, the common treatments used to control the progression are interferon beta, glatiramer acetate, mitoxantrone, and some monoclonal antibodies [8].

Some oral involvements in MS, like xerostomia and trigeminal neuralgia, have already been explored [9], but regarding the rarity of PCG, it has not been explored before. Given that both conditions involve aberrant immune activity, their coexistence raises important diagnostic and pathophysiologic considerations. This is a very unique condition, which is discussed as follows.

## 2. Case Presentation

A 26‐year‐old woman from Isfahan who has been under treatment for MS approached the Oral Medicine department with the chief complaint of gum bleeding and gingivitis and geographic tongue. She complains about the pain of gingiva and bleeding when brushing her teeth. She had been diagnosed with MS at the age of 17, initially presenting with blurred vision. Brain and spinal MRI revealed demyelinating plaques consistent with MS. She received interferon beta‐1a (Actovex) for 5 years, but treatment was discontinued due to severe vertigo, after which ocrelizumab (Xacrel) was initiated. She has experienced 2 MS relapses characterized by blurred vision and severe vertigo, both treated with intravenous prednisolone. She reported no recent changes in medications, oral hygiene products, or exposure to new substances, and she has a known allergy to pineapple.

She has not started with any new treatment and has not taken any new chemical or herbal drugs. She has also not changed her toothbrush or toothpaste (she has been using fluoride toothpastes from two brands, Merident and Signal). She works at a pharmacy and was not exposed to any new substance. She is only allergic to pineapples.

Her neurologist classifies her MS as relapsing‐remitting. She has no history of urinary or fecal incontinence. Previous fingertip paresthesia had resolved, and her only current neurological complaint is mild weakness. She does not smoke, consume alcohol, or use recreational drugs. Her medical history is otherwise notable only for vitamin D deficiency—treated with supplementation—and a previous episode of renal calculi. She is not an addict or smoker, and the patient reported no alcohol consumption. The only other medical condition is vitamin D deficiency, which has been treated with vitamin D pearls and a history of renal stones.

In the physical examination, she has normal neurological findings, her reflexes are in the normal range, and she has no sensory or motor dysfunction. While she experienced vertigo and nystagmus while gazing to the right 6 years ago, she does not have such symptoms now.

**Figure 1:**
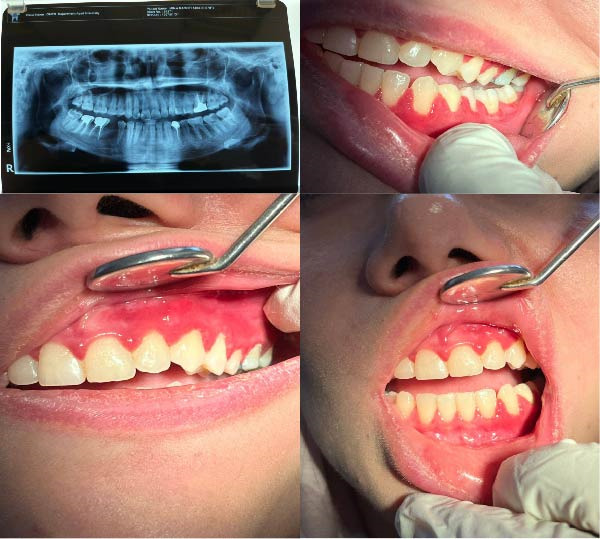
Clinical and radiographic pictures of the patient’s lesion.

In the oral examination, the gingiva was erythematous and tender, while she had good oral hygiene. Such changes are generalized but more severe on the left maxillary side. (Figure 1). Laboratory investigations were done, including biochemistry, hematological, and hormonal analysis and flow cytometry (Table 1). Reduced CD19 percentage is consistent with B‐cell depletion expected in patients receiving anti‐CD20 therapy such as ocrelizumab. Previous lab reports included a TSH of 2.8, an anti‐HCV nonreactive test, and an NMO‐antibody nonreactive test. For a definitive diagnosis, a biopsy was done on 20 October 2024 by taking a specimen from the gingiva, which was described by an expert pathologist.

**Table 1:** Lab findings.

	Test	Result	Reference interval for a 26‐year‐old woman
Biochemistry	Creatinine	0.68 mg/dL	0.6–1.2
	Blood urea nitrogen	9.2 mg/dL	7–18
	AST	12 U/L	<32
	ALT	15 U/L	<32

Hormone analysis	25‐OH‐Vitamin D	67.9 ng/mL	Sufficient: 30–100

Flow cytometry	CD19	0.01%	3–14

Hematology	WBC	6.8 10^3^/µL	4.0–10.0
	Lymphocytes	30.5%	25–50
	Neutrophils	63.7%	50–80
	Eosinophils	0.9%	0.0–2.5
	Hemoglobin	14.2 g/dL	12.5–15.5
	Platelets	287 10^3^/µL	150–450

The lesion involved all four quadrants of both the maxilla and mandible, presenting as an edematous, gingivitis‐like enlargement with a granular surface. A biopsy measuring 3 × 4 × 7 mm was obtained from the anterior maxilla adjacent to the upper left lateral incisor.

## 3. Microscopic Description

**Figure 2:**
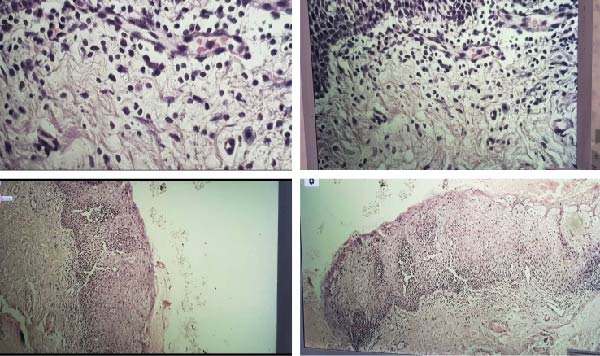
Microscopic view of the lesion biopsy.

Differential Diagnosis Encompassed Leukemia and Lymphoma. In the microscopic examination of multiple sections prepared from the gingival biopsy tissue, the surface epithelium is of squamous type and acanthotic. No dysplastic changes were observed in the surface epithelium. In the underlying stroma, an infiltration of inflammatory cells, including lymphocytes and plasma cells, is evident (Figure 2). These findings were compatible with PCG. To rule out leukemia, a peripheral blood smear was evaluated, and immunohistochemical studies were performed on the biopsy specimen to determine whether the plasma cell infiltration was monoclonal or polyclonal.

The patient was initially managed with chlorhexidine mouthwash, which resulted in partial clinical improvement. Due to the persistence of the lesion, a biopsy was subsequently performed. A corticosteroid mouthwash was eventually prescribed based on the histopathological findings and ongoing symptoms.

**Figure 3:**
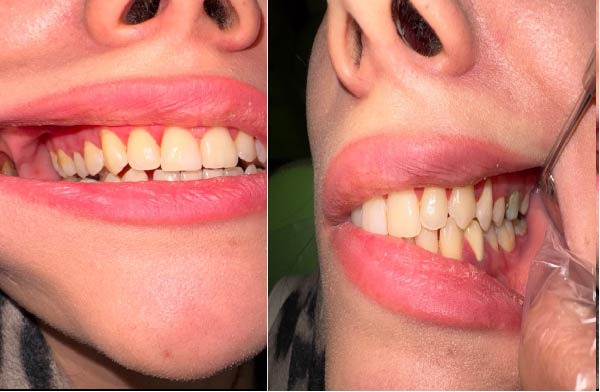
Clinical of the patient’s lesion after 6 months.

Magic mouthwash includes 4 AMP Betamethasone, syrup Diphenhydramine, and drop Nystatin 100,000 units mL, which are mixed three times a day for 1 month for PCG. Regarding the under‐control neurological symptoms, the previous treatment for MS was continued. At the 6‐month follow‐up visit, the patient reported both remission and intermittent episodes of recurrence during the recovery period. She stated that she had been prescribed chlordiazepoxide by her physician, which led to a noticeable reduction in anxiety. According to the patient, her oral manifestations improved in parallel with the decrease in anxiety levels. Upon reassessment, a corticosteroid mouthwash was prescribed again to manage the recurrent symptoms (Figure 3 and Table 2).

**Table 2:** Clinical timeline of the patient.

Time point	Age/week	Clinical event
Adolescence	17 years	Diagnosis of multiple sclerosis following blurred vision
Younge adulthood	17–22 years	Treatment with interfron beta‐1a
Younge adulthood	22 years	Switched to ocrelizumab due to vertigo
Adulthood	26 years	Onset of gingival pain and bleeding
Week 0	—	Initial oral examination and laboratory investigations
Week 1	—	Gingival biopsy performed
Week 2	—	Histopathological diagnosis of plasma cell gingivitis confirmed
Week 2	—	Initiation of topical corticosteroid mouthwash
Week 4	—	Follow‐up visit showing partial clinical improvement

## 4. Discussion

This report describes a rare case of a 26‐year‐old woman diagnosed with both MS and PCG, a combination not previously documented in the literature. The coexistence of these conditions presents a unique diagnostic and therapeutic challenge, as both involve immune dysregulation but manifest in distinct ways.

Both MS and PCG involve immune system malfunction. MS is characterized by central nervous system demyelination mediated by T cells, B cells, and macrophages and is considered an autoimmune disease, while PCG involves localized plasma cell infiltration in the gingival tissue due to hypersensitivity to antigens. The patient’s hypersensitivity to pineapples and the chronic inflammation in PCG suggest an overactive immune response. While no direct connection between MS and PCG has been established, cytokine imbalances or dysregulated immune surveillance may contribute to their coexistence.

Ocrelizumab, an anti‐CD20 monoclonal antibody, produces near‐complete depletion of circulating B cells. While plasma cells do not express CD20 and are not directly affected, B‐cell depletion may trigger compensatory immune mechanisms that increase plasma cell activity in mucosal tissues. Although speculative, this compensatory pathway could contribute to exaggerated plasma cell responses such as PCG in susceptible individuals.

Similar associations between PCG and other autoimmune or immune‐mediated diseases, including Crohn’s disease, psoriasis, and lupus erythematosus, have been reported. These connections support the concept that systemic dysregulation of immune responses may predispose patients to localized plasma cell‐predominant inflammatory conditions.

It is important to emphasize that this proposed mechanism remains a theoretical consideration and cannot be confirmed based on a single case report.

While MS has been associated with some oral health issues, including xerostomia and increased susceptibility to periodontal disease, no prior cases of PCG in MS patients have been reported. By documenting this dual diagnosis, this report not only contributes to the limited knowledge of how systemic autoimmune diseases might interact with localized oral inflammatory conditions but also raises the hypothesis that immune‐modulating therapies, such as ocrelizumab, may shift immune surveillance toward hypersensitivity reactions rather than infection, thereby predisposing susceptible individuals to exaggerated allergic responses like PCG. Further studies should investigate the potential role of such medications in modulating mucosal immune balance.

The diagnosis of PCG in this patient required careful differentiation from other gingival disorders that present with similar clinical features. such as lichen planus, autoimmune vesiculobullous disease, and drug eruptions. Also, the patient had an oral breathing habit, which could also cause gingival erythema and edema. A detailed history, laboratory investigations, and a biopsy were essential to rule out these conditions and confirm PCG.

Topical corticosteroids are considered the first‐line treatment for PCG because they effectively suppress plasma cell–mediated inflammation. In our patient, corticosteroid mouthwash led to a gradual reduction in gingival erythema, bleeding, and tenderness, consistent with previously reported therapeutic responses. This case highlights that topical corticosteroids remain essential for controlling PCG. Despite some symptom relief after anxiety reduction, recurrence at 6 months required represcription of a corticosteroid mouthwash, emphasizing its primary role in managing this chronic condition.

In evaluating potential contributors to her gingival condition, it was also important to consider whether her MS medications could have played a role. Regarding ocrelizumab, its hypersensitivity adverse effects are predominantly infusion‐related, and gingivitis has not been reported as an adverse effect. While there are more documented hypersensitivity reactions associated with interferon beta‐1a, gingivitis has not been listed as a side effect after long‐term follow‐up.

The absence of new medications or exposures before the onset of PCG supports the diagnosis of hypersensitivity‐induced gingivitis rather than a drug‐induced condition.

This case highlights the importance of considering rare oral conditions like PCG in patients with chronic autoimmune diseases such as MS. Routine oral health assessments in MS patients could facilitate early detection of unusual conditions. The case also emphasizes the role of collaboration between neurologists, dentists, and pathologists in achieving accurate diagnoses and effective management strategies.

A diagnostic limitation worth noting is the potential overlap between PCG and other plasma cell‐rich conditions such as plasma cell mucositis or lichenoid hypersensitivity reactions. However, the polyclonal nature of the infiltrate, together with clinicopathologic correlation, supported the diagnosis of PCG in this case.

A limitation of this report is the absence of long‐term follow‐up, which prevents assessment of sustained therapeutic response or recurrence. Ongoing periodic evaluations have been planned to monitor disease stability, particularly given the patient’s long‐term use of immunomodulatory therapy. Additionally, while the shared immunopathological mechanisms of MS and PCG are hypothesized, further research is needed to establish a definitive link.

## Funding

No funding was received for this manuscript.

## Consent

Written informed consent was obtained from the patient for publication of the clinical information and images. All the patients allowed personal data processing, and informed consent was obtained from all individual participants included in the study.

## Conflicts of Interest

The authors declare no conflicts of interest.

## Data Availability

The data that support the findings of this study are available upon request from the corresponding author. The data are not publicly available due to privacy or ethical restrictions.
